# The association between oxidative stress and corneal hysteresis in patients with glaucoma

**DOI:** 10.1038/s41598-020-57520-x

**Published:** 2020-01-17

**Authors:** Keiko Uchida, Noriko Himori, Kazuki Hashimoto, Yukihiro Shiga, Satoru Tsuda, Kazuko Omodaka, Toru Nakazawa

**Affiliations:** 1 Department of Ophthalmology, Tohoku University Graduate School of Medicine, Sendai, Japan; 2 Collaborative Program for Ophthalmic Drug Discovery, Tohoku University Graduate School of Medicine, Sendai, Japan; 3 Department of Ophthalmic Imaging and Information Analytics, Tohoku University Graduate School of Medicine, Sendai, Japan; 4 Department of Retinal Disease Control, Tohoku University Graduate School of Medicine, Sendai, Japan; 5 Department of Advanced Ophthalmic Medicine, Tohoku University Graduate School of Medicine, Sendai, Japan

**Keywords:** Biochemistry, Biomarkers, Risk factors

## Abstract

Systemic antioxidative status has been implicated in glaucoma pathogenesis. Additionally, corneal hysteresis (CH) may contribute to glaucoma progression. Here, we evaluated the relationship between biological antioxidant potential (BAP) and CH. This study included 103 patients with open-angle glaucoma (OAG). We used a free radical analyzer to measure BAP, and an ocular response analyzer to measure CH and corneal resistance factor (CRF). We evaluated the relationship between systemic oxidative stress and other clinical parameters with Spearman’s rank correlation test and a multi-regression analysis. BAP was not correlated to either CH or CRF in the male or female OAG patients. BAP was correlated to both CH and CRF in the female OAG patients older than 57 years (r = 0.51, *P* = 0.003; r = 0.49, *P* = 0.004), but uncorrelated in the female OAG patients younger than 57 years. Multiple regression analysis revealed that BAP independently contributed to CH (*P* = 0.025) and CRF (*P* = 0.015) in the older female OAG patients. Systemic oxidative stress may significantly affect the viscoelasticity of the cornea in older female OAG patients. Future studies are needed to confirm that low systemic antioxidative status and low corneal hysteresis contribute to glaucoma pathogenesis.

## Introduction

Patients with glaucoma undergo progressive visual field loss cause by retinal ganglion cell (RGC) death. Currently, treatments are used to lower intraocular pressure (IOP), slowing glaucoma progression^1^. However, lowering IOP is not always sufficient to prevent the development of optic neuropathy in glaucoma. Even when IOP is stable, optic nerve head damage can vary between individuals due to differing susceptibility to glaucomatous damage^2^. Some researchers have found that glaucoma progression is related to specific material properties of the cornea, such as the strength of corneal viscoelastic damping, known as corneal hysteresis (CH)^3,4^. CH can be measured by temporarily inducing a concave deformation in the cornea with a pulse of air. As the corneal surface is deformed and then returns to its normal shape, an ocular response analyzer (ORA) measures ocular pressure (in mmHg) at the two moments when the corneal surface is flat: when it first reaches applanation (P1) and when it bounces back (P2). The difference in pressure between these two points is defined as CH. Decreased CH is thought to reflect constriction originating in the extracellular matrix; it has been suggested that tissues at the back of the eye, including the peripapillary sclera and lamina cribrosa^5^, may also display hysteresis. Corneal resistance factor (CRF) is an indicator of the overall resistance of the cornea.

Glaucoma is thought to be a multifactorial disease related to ageing^6^, decreased ocular blood flow^7^, mitochondrial dysfunction^8^, and oxidative stress^9^. Our previous research has revealed that biological antioxidant potential (BAP), a systemic measure of antioxidative potential, is associated with disease severity in younger male glaucoma patients^10^. This prompted the current investigation of the effect of age on BAP and CH. We also investigated the influence of systemic parameters, including age and sex, on BAP, and determined whether BAP and CH were related to each in OAG. Therefore, this study sought to improve our understanding of the importance of systemic antioxidant capacity and oxidative stress in glaucoma.

## Results

### Clinical characteristics of the OAG patients

This study included 103 Japanese glaucoma patients (male: 45; female: 58, NTG: 82, POAG: 21). In a previous study, we divided patients into two groups based on mean age^10^. In this study, we divided the patients into four groups based on sex and mean age in each sex group (male: 61 years, female: 57 years). Table 1 shows demographic characteristics. The younger female and older female groups differed significantly in dROMs, axial length (*P* = 0.019, *P* = 0.033), and the incidence of hypertension and hyperlipidemia (*P* = 0.007, *P* = 0.001). There were no statistical differences between younger and older male patients with OAG.

**Table 1:** Characteristics of open angle glaucoma patients.

	Male				Female			
	All	<61 yrs	61 yrs≤	<61 yrs vs ≥61 yrs P value	All	<57 yrs	57 yrs≤	<57 yrs vs ≥57 yrs P value
n	45	23	22	—	58	26	32	—
NTG: POAG	32: 13	19: 4	13: 9	0.110*	50: 8	24: 2	26: 6	0.276*
Age (yrs)	60.8 ± 10.6	52.4 ± 5.9	69.6 ± 6.2	<0.001	56.7 ± 11.6	46.2 ± 6.2	65.3 ± 6.7	<0.001
dROM (U. Carr)	337.00 ± 54.38	339.78 ± 44.94	334.09 ± 63.74	0.874	391.78 ± 60.05	376.46 ± 64.78	404.22 ± 53.75	0.019
BAP (mmol/L)	2003.94 ± 270.53	2039.99 ± 249.66	1966.26 ± 291.77	0.395	2116.81 ± 234.00	2070.69 ± 231.87	2154.28 ± 232.57	0.458
MD (dB)	−14.71 ± 8.25	−12.41 ± 7.62	−17.12 ± 8.36	0.058	−11.22 ± 6.83	−9.94 ± 6.99	−12.26 ± 6.62	0.148
Axial length (mm)	25.51 ± 1.10	25.70 ± 1.20	25.32 ± 0.97	0.229	24.96 ± 1.18	25.31 ± 1.20	24.67 ± 1.11	0.033
GAT (mmHg)	14.13 ± 3.34	14.38 ± 3.54	13.86 ± 3.18	0.680	14.45 ± 3.04	14.92 ± 2.70	14.36 ± 3.03	0.550
CH (mmHg)	9.86 ± 1.26	10.05 ± 1.37	9.66 ± 1.17	0.312	10.05 ± 1.41	9.96 ± 1.54	10.13 ± 1.31	0.537
CRF (mmHg)	9.30 ± 1.68	9.71 ± 1.76	8.87 ± 1.51	0.120	9.19 ± 1.87	9.13 ± 1.96	9.23 ± 1.83	0.857
CCT (mm)	514.91 ± 35.79	520.13 ± 36.06	509.45 ± 35.52	0.388	506.22 ± 34.26	506.77 ± 36.45	505.78 ± 32.96	0.981
Hypertension (%)	17 (37.78)	6 (26.09)	11 (50.00)	0.130*	12 (20.69)	1 (3.85)	11 (34.38)	0.007*
Diabetes (%)	6 (13.33)	2 (8.70)	4 (18.18)	0.414*	4 (6.90)	0 (0.00)	4 (12.50)	0.120*
Hyperlipidemia (%)	14 (31.11)	4 (17.39)	10 (45.45)	0.057*	11 (18.94)	0 (0.00)	11 (34.38)	0.001*
Current smoker (%)	6 (13.33)	4 (17.39)	2 (9.09)	0.665*	3 (5.17)	2 (7.69)	1 (3.13)	0.582*

NTG = normal tension glaucoma, POAG = primary open angle glaucoma, MD = mean deviation, dROM = diacron reactive oxygen metabolites, BAP = biological antioxidant potential, U. Carr = Carrelli units, GAT = Goldmann applanation tonometry, CH = corneal hysteresis, CRF = corneal resistance factor, CCT = central corneal thickness.

Unmarked *P* values: Mann-Whitney U test, ^*^Fisher exact test.

### Association between CH or CRF and BAP

**Figure 1:**
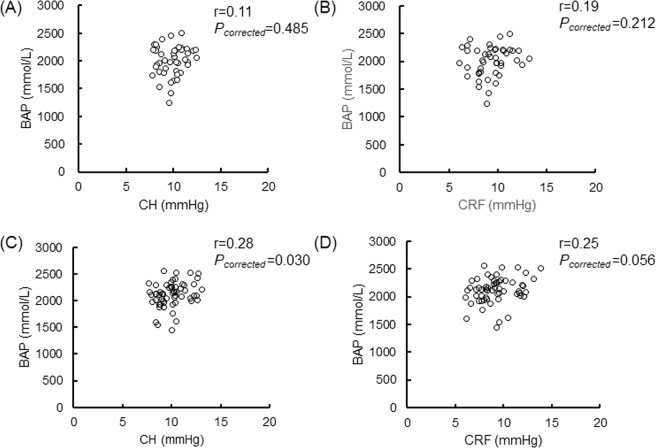
Relationship between BAP and CH or CRF in various age and sex groups. Scatterplots showing the relationship between BAP and CH in (**A**) all male subjects, (**C**) all female subjects, between BAP and CRF in (**B**) all male subjects, and (**D**) all female subjects. Correlations were not found in A (r = 0.11, *P*_*corrected*_ = 0.485), B (r = 0.19, *P*_*corrected*_ = 0.212), C (r = 0.28, *P*_*corrected*_ = 0.030) or D (r = 0.25, *P*_*corrected*_ = 0.056). Differences were considered significant at *P*_*corrected*_ < 0.015 (0.05/4). BAP = biological antioxidant potential, CH = corneal hysteresis, CRF = corneal resistance factor.

**Figure 2:**
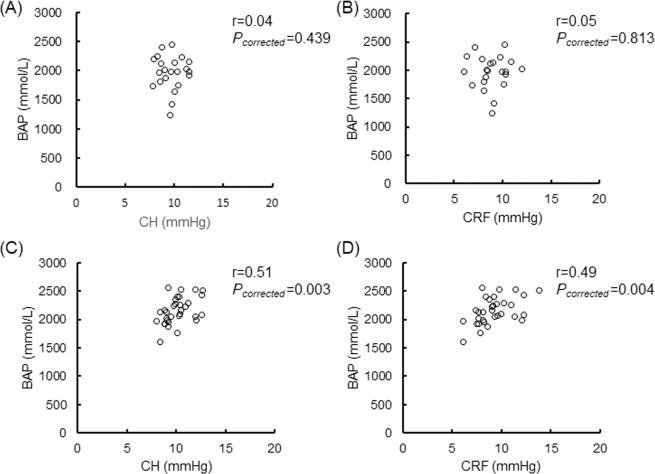
Relationship between BAP and CH or CRF in older male and female subjects. Scatterplots showing the relationship between BAP and CH in (**A**) older male subjects, (**C**) older female subjects, between BAP and CRF in (**B**) older male subjects, and (**D**) older female subjects. Correlations were not found in A (r = 0.04, *P*_*corrected*_ = 0.439) or B (r = 0.05, *P*_*corrected*_ = 0.813). Correlations were found in C (r = 0.51, *P*_*corrected*_ = 0.003) and D (r = 0.49, *P*_*corrected*_ = 0.004). Differences were considered significant at *P*_*corrected*_ < 0.015 (0.05/4). BAP = biological antioxidant potential, CH = corneal hysteresis, CRF = corneal resistance factor.

**Figure 3:**
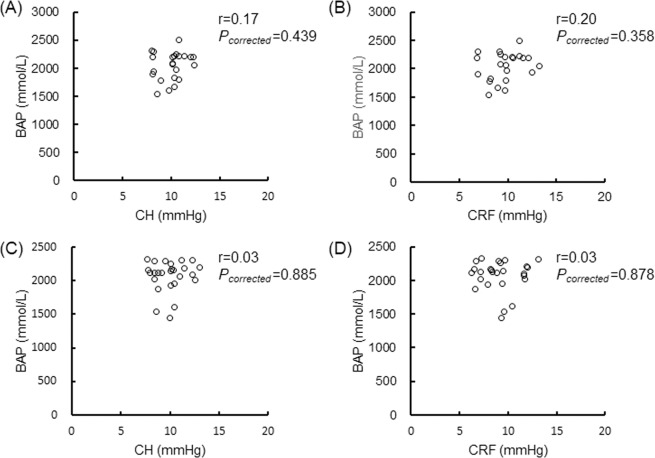
Relationship between BAP and CH or CRF in younger male and female subjects. Scatterplots showing the relationship between BAP and CH in (**A**) younger male subjects, (**C**) younger female subjects, between BAP and CRF in (**B**) younger male subjects, and (**D**) younger female subjects. Correlations were not found in A (r = 0.17, *P*_*corrected*_ = 0.439), B (r = 0.20, *P*_*corrected*_ = 0.358), C (r = 0.03, *P*_*corrected*_ = 0.885) or D (r = 0.03, *P*_*corrected*_ = 0.878). Differences were considered significant at *P*_*corrected*_ < 0.015 (0.05/4). BAP = biological antioxidant potential, CH = corneal hysteresis, CRF = corneal resistance factor.

BAP was not correlated with either CH or CRF in the male or female patients (Fig. 1A–D). We found that BAP was positively correlated to CH and CRF in the female OAG patients older than 57 years (r = 0.51, *P*_*corrected*_ = 0.003; r = 0.49, *P*_*corrected*_ = 0.004; Fig. 2C,D), but not correlated in the female OAG patients younger than 57 years (Fig. 3C,D). There were no significant relationships in the younger or older male patients (Figs. 2A,B and 3A,B).

### Multiple regression analysis

A univariable regression analysis revealed that age, BAP, GAT, CCT and hyperlipidemia were independent contributing factors to CH in female OAG patients older than 57 years (*P* = 0.007, *P* = 0.008, *P* = 0.001, *P* < 0.001, *P* = 0.010; Table 2A). There was a positive correlation between GAT and CCT. The regression coefficient (β) was higher in CCT than GAT, prompting us to choose CCT for further investigation. The regression coefficient (β) of hyperlipidemia was the lowest among the variables. We selected three variables: age, BAP, and CCT. We applied a multiple regression analysis, which showed that BAP, age and CCT remained independent contributing factors to CH (*P* = 0.025, *P* = 0.048, *P* < 0.001; Table 2A). Univariable regression analysis revealed that BAP, GAT and CCT were independent contributing factors to CRF in female OAG patients older than 57 years (*P* = 0.005, *P* < 0.001, *P* < 0.001; Table 2B).

**Table 2:** Factors contributing to CH and CRF in female OAG patients older than 57 years.

Variable	Univariable model			Multivariable model		
	β	(95% CI)	P value	β	(95% CI)	P value
**(A)**						
Age (yrs)	0.47	0.0273 to 0.1561	0.007	0.22	0.0005 to 0.0842	0.048
LogMAR	−0.01	−2.4069 to 2.2992	0.963			
dROM (U. Carr)	−0.11	−0.0117 to 0.0064	0.553			
BAP (mmol/L)	0.46	0.0007 to 0.0045	0.008	0.24	0.0002 to 0.0026	0.025
GAT (mmHg)	0.57	0.1147 to 0.3907	0.001			
CCT (mm)	0.79	0.0223 to 0.0406	<0.001	0.65	0.0172 to 0.0348	<0.001
Axial length (mm)	−0.22	−0.6901 to 0.1718	0.229			
cpRNFLT (mm)	0.04	−0.0323 to 0.0397	0.834			
MD (dB)	0.08	−0.0572 to 0.0903	0.650			
Hypertension	−0.17	−0.7269 to 0.2547	0.334			
Hyperlipidemia	−0.44	−1.0479 to −0.1535	0.010			
**(B)**						
Age (yrs)	0.35	−0.0008 to 0.1892	0.052			
LogMAR	0.06	−2.7012 to 3.8261	0.727			
dROM (U. Carr)	−0.17	−0.0182 to 0.0067	0.352			
BAP (mmol/L)	0.49	0.0012 to 0.0063	0.005	0.31	0.0005 to 0.0044	0.015
GAT (mmHg)	0.64	0.2141 to 0.5704	<0.001			
CCT (mm)	0.73	0.0260 to 0.0544	<0.001	0.64	0.0286 to 0.0491	<0.001
Axial length (mm)	−0.11	−0.7987 to 0.42095	0.532			
cpRNFLT (mm)	0.00	−0.0501 to 0.0502	0.998			
MD (dB)	−0.02	−0.1074 to 0.0983	0.929			
Hypertension	−0.05	−0.8069 to 0.6038	0.771			
Hyperlipidemia	−0.33	−1.3057 to 0.027	0.060			

VA = visual acuity, logMAR = logarithm of minimum angle of resolution, dROM = diacron reactive oxygen metabolites, BAP = biological antioxidant potential, U. Carr = Carrelli units, GAT = Goldmann applanation tonometry, CCT = central corneal thickness, MD = mean deviation, cpRNFLT = circumpapillary retinal nerve fiber layer thickness, β = standard partial regression coefficient.

Multiple regression analysis revealed that BAP and CCT remained independent contributing factors to CRF (*P* = 0.015, *P* < 0.001; Table 2B) in the older group of female OAG patients.

## Discussion

Our findings suggest that systemic antioxidative status may significantly affect the viscoelasticity of the cornea in female OAG patients older than 57 years. In a previous study, we found that sex and age interacted with systemic antioxidative status to influence the pathogenesis of glaucoma and glaucomatous damage^10^. This prompted our decision to separately evaluate male and female groups, as well as older and younger, groups. Other studies have already demonstrated that low CH is associated with glaucoma progression^3,4^. Here, we evaluated the relationship between systemic antioxidative status and CH; to the best of our knowledge, this is the first study to do so. This study had two underlying hypotheses: first, that systemic antioxidative status may contribute to glaucoma pathogenesis by affecting CH; and second, that relatively older female patients with a low antioxidant level might be more susceptible to oxidative stress, resulting in glaucoma.

Collagen, keratocytes, and proteoglycans are the major components of the corneal stroma. Collagen is secreted by keratocytes, and eventually degraded by matrix metalloproteinases (MMPs). The collagen in the corneal stroma is continuous with that in the sclera and lamina cribrosa. Thus, it is likely that the cornea, sclera, and lamina cribrosa share similar biomechanical characteristics, and CH may represent an indirect measure of lamina cribrosa compliance^5^.

A variety of factors may modulate the balance between corneal collagen production and degradation. Oxidative stress accelerates the apoptosis of keratocytes, while hypoxia reduces collagen expression^11^. Moreover, oxidative stress and the nitric oxide pathway reduce the activity of tissue inhibitor of metalloproteinases (TIMP)-1 and MMP-2, resulting in destruction of the extracellular matrix^12^. Collagen cross-linking induced by oxidative stress increases corneal rigidity and collagen fiber thickness^13^. In this study, we found a significant difference between the younger and older female OAG patients in dROMs. High oxidative stress results in decreased collagen, which in turn enhances the compliance of the lamina cribrosa, yielding a worse environment for the ONH and leading to RGC death.

One of the distinguishing characteristics of relatively older female patients is low estrogen levels, a hormone known to have a neuroprotective effect in glaucoma. Importantly, studies have demonstrated that estrogen in the eye has a strong effect on the growth of collagen fibers, which are an important supportive tissue in the extracellular matrix of the lamina cribrosa. It has also been reported that CCT increases and decreases during the menstrual cycle with the estrogen level^14^. Zhou *et al*. investigated the effects of sex hormones on collagen degradation and MMP expression by corneal fibroblasts^15^. They found that 17β-estradiol inhibited collagen degradation by the corneal fibroblasts through down-regulation of the expression and activity of MMPs. Wickham *et al*. revealed the presence of estrogen receptors in the cornea^16^. These findings suggest that estrogen has a protective effect against glaucoma, acting by increasing collagen content and inducing an IOP decrease. The increased collagen also enhances the biomechanical properties of the lamina cribrosa, resulting in a better environment for the ONH connective tissues. Additionally, female hormones have an antioxidative effect. We previously found that in younger male patients, there was a relationship between reduced BAP and glaucoma severity, but that this relationship was absent in younger female patients^10^. However, after menopause, female subjects with low BAP are susceptible to changes in CH. Thus, abundant evidence suggests that estrogen has an influence on collagen fiber content in the eye and thereby contributes to many disease mechanisms.

Tanito *et al*. found that there was no significant difference in dROMs between control and glaucoma groups^17^. In the current study, there were no control subjects, but in a previous study, we found that there were no significant differences in dROMs between controls and any of several OAG subgroups^10^. In the current study, moreover, there was a significant association between dROMs and age in the female OAG patients, but not in the male OAG patients. Thus, oxidative stress, particularly dROM levels, may have depended on age in the female OAG patients in this study. As it is still difficult to evaluate systemic imbalances between ROS and antioxidant levels, we consider that BAP has great potential for future use as a predictive biomarker of oxidative stress-induced glaucomatous damage, particularly under circumstances when the production of ROS is normal.

Low systemic antioxidative capacity lowers the ability to defend against oxidative stress, which leads to oxidative damage and higher disease severity in glaucoma^10^. One of the most important antioxidants is glutathione. A recent clinical study of glaucoma showed that low glutathione levels were associated with higher susceptibility to glaucoma^18^. Nitric oxide synthase is also an important enzymatic antioxidant. Ingested nitrate is turned into nitric oxide, a vasodilator that improves ocular blood flow. Kang *et al*. showed that a diet high in dark green leafy vegetables, which are a source of nitrate, had a significant protective effect in OAG^19^. This suggests that oxidative stress may contribute to glaucoma by reducing the level of serum anti-oxidant proteins. The main addition of the current study to previous reports is the finding that there is a close association between systemic antioxidant levels and CH only in relatively older female patients with OAG. Thus, this subset of patients might benefit from anti-oxidant therapy.

The present study has several limitations. First, we did not measure the levels of female hormones. This might have provided useful data, because we found an association between BAP and CH in the female glaucoma patients older than 57 years, and the average age of menopause in Japan is 48.3 ± 4.7 YO^20^. Thus, the patients older than 57 years were likely postmenopausal and had a low level of female hormones. Second, somewhat paradoxically, we found that CH seemed to improve with age in the female glaucoma patients older than 57 years. It is possible that other, unknown pathways might compensate for changes in CH under low levels of female hormones. Third, BAP has been found to be associated with the level of uric acid, a marker of renal function^21,22^. Tanito *et al*. found that BAP was a good indicator of the functional effects of OAG, and reported an association between lower BAP and higher IOP in OAG^23^. Thus, measuring serum BAP may be a useful, reliable method of determining antioxidant capacity. We are planning to determine the association between glaucoma and directly measured antioxidant levels in a future study. Forth, there was a general association between dROMs and BAP. Along with an increased dROM level, BAP tended to increase in the female OAG patients older than 57 years compared to the younger female OAG patients. There were no other significant differences between the younger and older female OAG patients. Finally, the number of OAG patients was relatively small. Future, larger-scale investigations are thus required.

It is already known that alterations in IOP do not affect every eye in the same way. For example, the EMGT and the Collaborative Normal-Tension Study reported that progression still occurred in some eyes with NTG despite medically-lowered IOP^1^. Our study contributes to previous evidence that low CH is a risk factor for glaucomatous damage even when IOP is in the normal range^24^. Furthermore, CH might be a surrogate biomarker of the biomechanical properties of tissues located in the posterior of the eye, such as the lamina cribrosa^25^. Finally, we found that older female subjects were more susceptible to low CH and high-IOP-induced glaucomatous damage, likely because female hormone levels and systemic antioxidant potential are low in postmenopausal women. Thus, this study supports the view that low BAP and low CH are risk factors for glaucoma.

## Methods

### Subjects

This retrospective study comprised a consecutive series of 103 Japanese patients who had previously been diagnosed with OAG. All patients were already undergoing treatment with antiglaucoma drugs. All subjects were examined at the ophthalmology clinic of Tohoku University Hospital during a period starting in April 2017 and ending in April 2018. Study protocols followed the Declaration of Helsinki and were approved by the Tohoku University School of Medicine Ethics Committee (study 2017-1-290). All participants provided informed consent. Data on the presence or absence of hyperlipidemia, hypertension, diabetes, and smoking habits were obtained.

### Clinical parameters

All patients received an ophthalmic evaluation that included best-corrected visual acuity (logMAR: logarithm of the minimal angle of resolution) and axial length. Intraocular pressure (IOP) was measured using Goldmann applanation tonometry (GAT). Mean deviation (MD) was measured with the Humphrey field analyzer (HFA; Carl Zeiss Meditec, Dublin, CA; Swedish interactive threshold algorithm (SITA)-standard strategy of the 24-2 program. Anterior-segment optical coherence tomography (AS-OCT; Casia, Tomey Co., Nagoya, Japan) was used to measure central corneal thickness (CCT). The subjects also underwent cpRNFLT measurement with conventional OCT (3D-OCT 2000; Topcon Co., Tokyo, Japan). A glaucoma specialist evaluated the optic disc in each patient using a 90-diopter lens. Exclusion criteria were the presence of other eye disease, including trauma, pigment dispersion glaucoma, angle-closure glaucoma, exfoliative glaucoma, secondary glaucoma, or strong myopia (i.e., axial length above 26.5 mm). The statistical analysis used the eye with worse MD.

### Corneal hysteresis measurement

ORA (Reichert Ophthalmic Instruments Inc, Depew, New York, USA) was used to measure CH, corneal-compensated IOP (IOPcc), corneal resistance factor (CRF), and Goldmann-correlated IOP (IOPg). Trained examiners measured each eye 3 times. Three measurements were averaged for use in the analysis. The quality of ORA measurements is determined based on a waveform score produced by the device. Measurements with a score of at least 6.5 were included. Details of these techniques have been reported previously^4,26^.

### Blood samples and testing

Blood samples were collected after at least 3 hours of fasting. A free radical analyzer (Free Carpe Diem, Wismerll Co., Ltd., Tokyo, Japan) was used to measure dROM and BAP. DROMs reflect oxidative stress levels and are an indicator of the serum activity of hydroperoxides. The BAP of a sample reflects its antioxidant potential and comprises its capacity to reduce ferric oxide to ferrous oxide. Details of these analysis methods have been described previously^10^. This method is the same as that used in previously published reports, and is routinely used to measure oxidative stress levels^17,23^.

### Statistical Analysis

This study used Spearman’s rank correlation test to determine single correlations between variables in each of the four groups (male/female, younger/older). Mann-Whitney U test or Fisher’s exact test were used to compare groups. We performed simple and multiple regression analyses to investigate variables independently affecting CH or CRF. Data are the mean ± standard deviation. We defined statistical significance as *P* < 0.05. Bonferroni corrected *P* values were used, to compensate for the effect of multiple testing (*P*_corrected_). Differences were considered significant at *P*_corrected_ < 0.015 (0.05/4 groups) for Spearman’s rank correlation test. JMP Pro 11 software was used for all statistical analyses (SAS Institute Japan, Inc., Tokyo, Japan).
